# Taking two to tango: fMRI analysis of improvised joint action with physical contact

**DOI:** 10.1371/journal.pone.0191098

**Published:** 2018-01-11

**Authors:** Léa A. S. Chauvigné, Michel Belyk, Steven Brown

**Affiliations:** Department of Psychology, Neuroscience & Behaviour, McMaster University, Hamilton, ON, Canada; Radboud Universiteit, NETHERLANDS

## Abstract

Many forms of joint action involve physical coupling between the participants, such as when moving a sofa together or dancing a tango. We report the results of a novel two-person functional MRI study in which trained couple dancers engaged in bimanual contact with an experimenter standing next to the bore of the magnet, and in which the two alternated between being the leader and the follower of joint improvised movements. Leading showed a general pattern of self-orientation, being associated with brain areas involved in motor planning, navigation, sequencing, action monitoring, and error correction. In contrast, following showed a far more sensory, externally-oriented pattern, revealing areas involved in somatosensation, proprioception, motion tracking, social cognition, and outcome monitoring. We also had participants perform a “mutual” condition in which the movement patterns were pre-learned and the roles were symmetric, thereby minimizing any tendency toward either leading or following. The mutual condition showed greater activity in brain areas involved in mentalizing and social reward than did leading or following. Finally, the analysis of improvisation revealed the dual importance of motor-planning and working-memory areas. We discuss these results in terms of theories of both joint action and improvisation.

## Introduction

Humans routinely engage in joint actions, where several individuals coordinate their behaviors around a common goal, generally for cooperative purposes, for example the members of a rowing team rowing in synchrony [1,2]. Joint actions can vary with regard to whether the individuals are all performing the same action (a bucket brigade) or complementary actions (tango dancers). For the purposes of the present study, our principal interest relates to the differential roles that partners can play during joint actions, with an emphasis on the contrast between leaders and followers. At one extreme are situations in which the role of each individual is explicitly defined and is maintained throughout the course of the action, for example during many couple dances or in ensemble musical performances. At the other extreme are situations where there may be no differences in the roles of the participants, for example during a bucket brigade, where each person’s movement pattern is fixed and repetitive, and where every person performs the same movement. In between, are situations where leadership is fluid–rather than being either fixed or absent–as in the turn-taking that occurs during conversation or the complementary pushing and pulling actions that take place when moving a piece of furniture.

An important characteristic of these roles is the degree to which an individual adapts to one’s partner(s) during the course of the interaction, in other words “who adapts to whom and to what degree” [3] (p. 688). A person who adapts to others more than others adapt to him/her is considered a follower. A follower focuses on the external sensory cues that allow the person to align his/her behavior both spatially and temporally to the behavior of his/her partners. Because the actions of a follower are dependent on others’ actions, following can be seen as an externally-driven behavior. Leading, in contrast to this, is far more internally-driven. A leader is less adaptive to others, and therefore mainly acts in accordance with his/her own intentions [3]. A leader is often the initiator of joint actions and is the major determinant of the spatio-temporal characteristics of the movements. As a result, leaders have to devise strategies to communicate their intentions to followers in order to accomplish the goals of the group [4,5]. Leading and following should not be seen as dichotomous roles, but instead as a continuum that depends on the degree to which each partner adapts to others [3]. Even for a pair of dancers or a group of musicians, where leadership roles are explicitly defined, partners nonetheless adapt to one another in an ongoing and mutual manner [6–8].

To the best of our knowledge, the only functional magnetic resonance imaging (fMRI) study to explicitly examine the dynamics of leading and following was that of Fairhurst et al. [3], in which participants performed an auditory finger-tapping task not with a human partner but with a computer program in the form of a virtual adaptive partner. The authors identified post-hoc those participants who tended to lead, compared to those who tended to follow, with leaders being defined as individuals who had a greater self-focus and who prioritized a stable tempo for the task, and followers as individuals who had a greater other-focus and who prioritized synchronization with their partner over stability of the task. Leaders showed greater activity in brain areas involved in self-initiated action, such as the pre-supplementary motor area (pre-SMA), dorsal anterior cingulate cortex (dACC), premotor cortex (PMC), right inferior frontal gyrus (IFG), and right inferior parietal lobule (IPL), as well as areas related to the integration of information coming from the self and other, such as the precuneus. However, Fairhurst et al. [3] did not report any activity specific for the followers.

A handful of electroencephalography (EEG) studies have looked at the neural basis of leading and following using hyperscanning methods. Sänger et al. [9], in a study of guitar duetting, showed that the pre-assigned leaders of these duets had an increased phase locking in the delta frequency range before playing onsets and a more distributed network of activity than did followers, which may reflect, respectively, the decision to initiate playing and a greater sense of effort. Konvalinka et al. [10] examined a situation of joint finger tapping between two people, and demonstrated that spontaneously-emerging leaders, who adapted less to their partner during the task, had frontal alpha suppression related to an increase in resources allocated for self-processing, indicating a strategy to focus on their own taps. Leading thus seems to rely on motor processing and the self-initiation of action.

Studies of interactive imitation of visually-presented hand movements, in which one participant performs a movement that should be imitated by another, explicitly assign participants the roles of leader or follower [11–13]. Performing an action to be imitated by another person engages a set of brain areas similar to the profile of a leader observed by Fairhurst et al. [3], whereas imitating someone else’s actions enhances activity in occipito-parietal regions, which may reflect enhanced attention to visual information [12] and/or the integration of visual information in order to elaborate a motor plan and regulate movement [13]. One might expect that following through another sensory modality (e.g., auditory or tactile) would lead to an increased reliance on the modality-related sensory-processing areas. In addition, interactive tasks of all kinds engage social-cognitive processes and hence activate areas associated with the mentalizing network, including the posterior superior temporal sulcus (pSTS), temporo-parietal junction (TPJ), and medial prefrontal cortex (mPFC) [14–16].

The efficacy of leading and following depends on an exchange of information between partners, as related to both the conveyance and perception of leadership cues. While the above-mentioned studies focused on the use of auditory and visual cues for guiding coordination, far less is known about the exchange of haptic information, which is the focus of the present study. This is surprising considering the strong involvement of haptic cues in real-world joint action, such as when moving furniture together or dancing with a partner. In these settings, force-based cues (i.e., pushing and pulling forces), perceived either through direct body contact or through the intermediary of a jointly-handled object, create a haptic information channel that allows individuals to coordinate their actions [17]. For example, during a partnered dance, the leader uses his arms to exert forces on the follower’s upper body in order to signal his movement intentions. The haptic channel may provide a particularly efficient mechanism for interpersonal coordination, since humans entrain more effectively with one another through tactile than auditory coupling [18], although other modalities may become more explicit with training [19]. This might be due to the faster response-time of haptic than auditory or visual stimulation [20] and to the higher coupling strength of haptic contact, which supports both informational (sensory) and mechanical (physical) coupling. Mechanical links exert a stronger coupling over a system, which enhances joint coordination [17,21–23]. Moreover, haptic stimulation is less affected by the degree of attentional engagement [24], and so it can be a spontaneous source of interpersonal coordination during joint action. In spite of the prominent role of haptic information transmission during everyday interactions, the neuroscience of joint action with haptic contact has been poorly studied.

Another important feature related to the differentiation of roles during joint action is the extent to which the actions of the individuals are fixed or are improvised. Situations where the action patterns are not pre-specified and must be created in the moment tend to foster a differentiation of roles into leaders and followers [5,25]. Motor improvisation involves the online generation of novel motor sequences, as guided by movement planning, often from pre-learned repertoires of movement sequences. It also includes the use of sensory feedback to modify ongoing production [26–28]. There have been a number of studies that have looked at motor improvisation at the individual-subject level, mainly work on musical improvisation, which tends to compare improvised performance of musical sequences with the execution of pre-learned sequences, for example comparing the improvisation of jazz sequences with the performance of musical scales [29]. Such studies have revealed the importance of two types of brain areas for improvisation, namely motor-planning areas (domain-specific) and working memory areas (domain-general). The former include the PMC, pre-SMA, IPL, and IFG, whereas the latter include the dorsolateral prefrontal cortex (DLPFC) and dACC [26–28,30–32] (see also the meta-analyses of [33,34]). Interestingly, studies of willed action and pseudorandom generation of responses have highlighted a network highly similar to the ones just mentioned [28,30,35]. In the present study, we were interested in seeing if the brain network that underlies solo improvisation during transitive tasks like piano performance would be recruited during a *joint* task involving direct body contact between two individuals, something similar to a dance improvisation. In addition, the present neuroimaging study is one of the first attempts to examine the joint improvisation of a motor task. Donnay et al. [31] demonstrated that interactive musical improvisation with a partner engaged more neural resources for action monitoring and working memory than performing a pre-learned interactive task. However, this study did not compare joint improvisation with solo improvisation.

In order to explore for the first time the neural basis of leading and following in a situation of joint improvisation with direct physical contact–akin to a couple dance–we carried out a novel 2-person fMRI experiment using highly trained couple dancers (e.g., tango, salsa) as participants. In the experiment, the participant engaged in bimanual contact with an experimenter standing next to the bore of the magnet so that the two could generate joint motor actions. In different conditions, the participant acted as either the leader or the follower of the joint hand movements, all done with the eyes closed and without any type of acoustic timekeeper (e.g., music) so as to limit communication to haptic interactions. In these conditions, the movement patterns were improvised, rather than pre-learned, in order to maintain an ongoing requirement for motor planning during leading and a comparably heightened sense of responsiveness to force-cues during following. The major goal of the experiment was to identify the neural signatures of leading and following in a situation of joint action with physical contact. In order to look at joint action in the absence of the leader/follower asymmetry, we created a “mutual” condition in which the participant and experimenter performed a pre-learned (rather than improvised) motor pattern with symmetrical roles, such that the conveyance and reception of forces were roughly comparable between the two actors. This allowed us to compare complementary vs. symmetric interactions. Finally, as a control for the motor requirements of the partnered conditions, we had participants perform a “solo” condition of improvised bimanual movements, but in the absence of physical contact with the experimenter.

We hypothesized that 1) partnered movements, compared to solo movements, would activate brain circuits involved in somatosensory and proprioceptive functioning, as well as social-cognition areas associated with interpersonal interaction, 2) leading would be associated with a motor network, including brain areas involved in motor planning, navigation, and self-initiated action, 3) following would be associated with a haptic sensory network that would mediate responsivity to haptic cues, 4) the brain network for mutual interaction would be intermediate between leading and following, 5) dance-like movement improvisation would engage a network similar to that established from other domains of motor improvisation, and 6) improvising within the context of joint action, as compared with solo improvisation, would be associated with increased demands on working memory and motor planning.

## Methods

### Participants

Nineteen dancers participated in the study after giving their written informed consent. The data from one participant was excluded due to excessive head movement. Of the remaining eighteen participants (9 males, mean age 40.4 ± 13.2), two were left-handed (1 male, 1 female). All were experts in one or several types of couple dances involving leading and following (Argentine Tango, Salsa, Swing, Ballroom), with a mean experience of 8.7 ± 7.2 years for males and 5.6 ± 2.9 years for females. None of the participants had a history of neurological or psychiatric disease. Participants received monetary compensation for their time. The study was approved by the Medical Research Ethics Board of St. Josephs Hospital, Hamilton, Canada (approval number: R.P. #12–3777).

### Procedure

**Fig 1:**
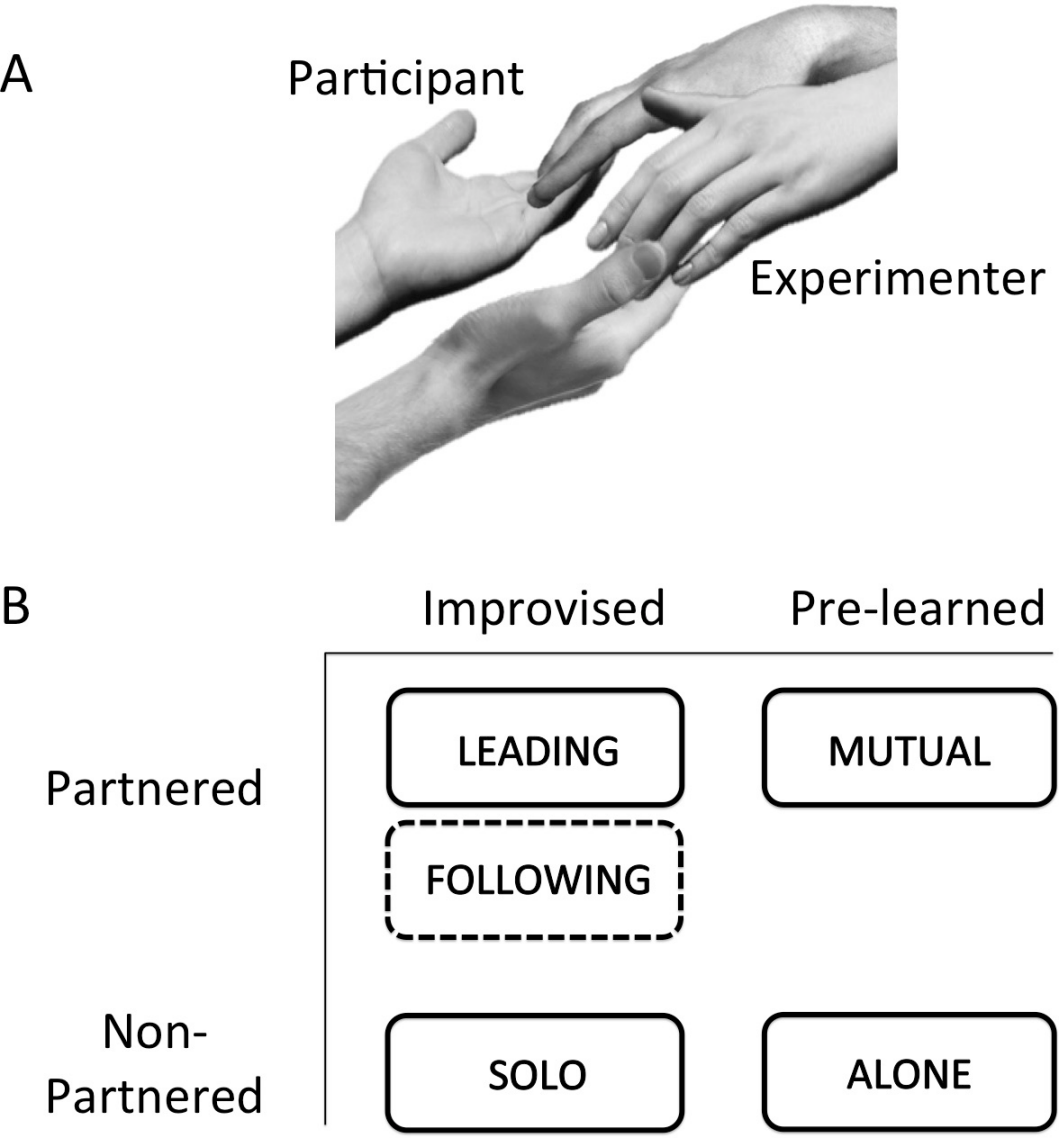
Experimental design. (A) This figure shows the contact between the participant and experimenter during the partnered conditions. They performed bimanual movements of the wrist and fingers in all three planes of motion, with principal contact occurring at the inner surfaces of the fingers. The participant’s hands were always palm-up below the experimenter’s hands. (B) The tasks were organized according to a 2 x 2 scheme, where one variable was partnership (partnered vs. solo tasks) and the other was improvisation (improvised vs. pre-learned movement patterns). There were five movement conditions and a baseline condition of Rest (not shown in the figure). The tasks of interest were the partnered conditions of Leading, Following, and Mutual. As a control for partnering, we had participants perform similar motor tasks, but on their own (Solo and Alone). Regarding the improvisation variable, the movements during Leading, Following and Solo were improvised. During the two non-improvised conditions, namely Mutual (partnered) and Alone (non-partnered), participants performed pre-learned movement sequences. Note that the Following condition did not tap into improvisational mechanisms of production on the part of the participant, as indicated by the jagged line for Following in the figure. Hence, the Following condition was excluded in the analysis of the main effect of improvisation.

While a participant was lying supine in the MRI scanner, an experimenter (female, with 8 years of couple dance experience, L.A.S.C.) stood next to the bore of the scanner so as to be able to engage in bimanual contact with the participant. The side of the experimenter was counterbalanced across scans and participants. Together, the participant and experimenter performed highly controlled bimanual movements of the wrist and metacarpophalyngeal joints in all three planes of motion, with principal contact occurring at the inner surfaces of the fingers (Fig 1A). The participant’s hands (palms up) were always below the experimenter’s hands (palms down) such that his/her hands could not be passively moved by the experimenter; the participant had to actively move his/her hands in all conditions.

There were five movement conditions (Fig 1B) and a baseline condition of Rest. The tasks of interest were the partnered conditions of Leading, Following, and Mutual. As a control for partnering, we had participants perform similar movement tasks on their own (Solo and Alone) without contact with the experimenter. The movement patterns during Leading, Following and Solo were improvised, whereas they were pre-learned in Mutual and Alone. During Leading, the participant improvised the movements, which the experimenter followed. During Following, the experimenter improvised the movements, which the participant followed. Thus, the Following condition did not tap into improvisational mechanisms of production on the part of the participant. During Solo, the participant improvised on their own, employing the same kind of movement patterns that they would during Leading. Care was taken during a training session (see below) to ensure that the movements were globally matched among the three improvised conditions. Next, there were two non-improvised conditions, called Mutual (partnered) and Alone (non-partnered). During these conditions, participants performed one of three *pre-learned* movement sequences that were taught to them during a training session on a day prior to their scan. These sequences were designed to use the same joints and to match the degree of movement variation of the improvised conditions. The three sequences were randomized across the scans, where only one pattern was done per task-epoch. Whereas the participant and experimenter performed the Mutual condition in a partnered manner, neither of them acted as the leader or follower of the movement. Instead, the speed and amplitude of these fixed patterns arose from implicit mutual agreement. We opted for the Mutual condition to be pre-learned, rather than improvised (as in [36]), because it would have been difficult for us to verify that an improvised condition was indeed done mutually, rather than involving closely alternating bouts of leading and following between the two partners. Mutuality was more likely to emerge during pre-learned patterns where the participant and experimenter shared knowledge and goals. Finally, during Rest, participants were instructed to keep still and relax. All conditions were performed with they eyes closed and without music or auditory-entrainment cues in order to keep the focus on interpersonal entrainment through haptic cues, rather than on external entrainment to a musical beat. It should be pointed out that our paradigm has ecological relevance to dance, since many forms of contemporary dance and contact improvisation rely far more on haptic cues than on musical beats for coordination. Examples of the five movement conditions can be found in S1 Video.

All participants underwent a one-hour training session on a day prior to the scanning session while lying supine on a table. They were specifically instructed to 1) not move their neck, shoulders or elbows; 2) not move any of their fingers individually, but only do so together as a hand-unit (only the metacarpophalyngeal joints); 3) match the speed and movement variation across all conditions; and 4) be as creative as possible when improvising. The training session ended when participants were able to perform highly controlled movements that respected the above restrictions and when they had memorized the three movement sequences for the non-improvised conditions. During the actual scanning session, the experimenter (who saw every movement in parallel to the timing progression) took detailed notes between each scan to ensure that those conditions were always fulfilled.

During scanning, the participant’s head was firmly secured using foam pillows, and their forearms were fastened to the side of their body such that only their wrists, hands and fingers were able to move. Earplugs were used to help block out scanner noise. The participants and experimenter each wore MRI-compatible headphones and received verbal instructions through them. Participants were instructed to keep their eyes closed at all times. The tasks were performed according to a block design, alternating between 28s of task and 8s of a relaxation period that was excluded from the analysis. Toward the end of the relaxation period, a verbal auditory cue was delivered through the headphones informing the participant and experimenter of the next task to perform. Each task-epoch started with a high tone and ended with a low tone. All stimuli were presented using Presentation® software (version 14.4, www.neurobs.com). Each of the six tasks (five movement tasks and Rest) was performed six times in random order across three functional scans.

After scanning, participants were debriefed. They answered questions on a 5-point scale about the perceived difficulty of each task, an evaluation of their performance, the extent to which they had the feeling that they were dancing, and the extent to which they experienced auditory imagery of music while moving. These data were analysed using four one-way ANOVA’s, with five levels corresponding to each of the movement conditions, respectively.

One limitation of the present study is that we were unable to collect behavioral data on task performance in the scanner. This would have required technologies such as either MRI-compatible motion capture or electromyography that we did not have access to at our imaging facility. As mentioned above, the first author participated in all of the scanning sessions and was able to verify that task performance was done properly.

### Image acquisition

Axial T2*-weighted gradient-echo echo-planar images (EPI) with blood-oxygen-level-dependent (BOLD) contrast were acquired with a General Electric Achieva 3-Tesla MRI at the Imaging Research Centre at St. Joseph’s Hospital in Hamilton, Ontario. The imaging parameters were 2000 ms TR, 35 ms TE, 90° flip angle, 39 axial slices, 4 mm slice thickness, 0 mm gap, 3.75 mm × 3.75 mm in-plane resolution, 64 × 64 matrix, and 240 mm field of view effectively covering the whole brain and the cerebellum. An automatic shimming procedure was performed before each scan to minimize inhomogeneities in the static magnetic field. In order to avoid T1 saturation effects, we discarded the first four dummy volumes of each scan. For each of the three functional scans, 216 volumes–corresponding to 12 epochs of 28s task + 8s relaxation–were collected over 7’12”, leading to a total of 648 volumes. Intensive piloting and magnetic field (B0) testing showed no B0 distortion and very little susceptibility-by-movement distortion of the BOLD signal during this paradigm. Two magnetic field maps (5ms then 8ms TE) with the same imaging parameters as the EPI were also acquired in order to unwarp the EPI data. Structural images were acquired before the EPI sequences. The high-resolution structural images were T1-weighted (TR/TI/TE/flip angle = 7752 ms/450 ms/2.44 ms/12°, FOV = 240 mm, resolution = 320×194, slice thickness = 2.0 mm, in-plane voxel size = 0.75 mm × 1.25 mm, 164 sagittal slices).

### Image analysis

Functional and structural images were processed using BrainVoyager QX 2.8. Functional images were first spatially realigned and motion-corrected to the first volume of the first scan. Motion-correction analysis revealed that participants displayed very little head movement. For most participants (14 out of 18), translational and rotational corrections never exceeded 2 mm and 2°, respectively, across the three functional scans. Only three out of 54 scans (where total scans = 18 participants x 3 scans) were excluded because of motion that exceeded either 3 mm of translation or 3° of rotation. The mean and standard deviation of the relative framewise displacement [37] of each scan is reported in S1 Table. Following rigid motion correction, unwarping was performed with the relaxation method of “anatabacus”, a plugin in BrainVoyager [38] in order to correct for non-rigid deformations. A temporal high-pass filter was applied at a cut-off frequency of 0.0078 Hz, or 1/128 cycles. Three-dimensional spatial smoothing was performed using a Gaussian filter with a FWHM kernel size of 4 mm. Each functional scan was then normalized to the Talairach template [39]. The BOLD response for each task-block was modeled as the convolution of a 28s boxcar with a synthetic hemodynamic response function composed of two gamma functions. In a first-level fixed-effects analysis, beta weights associated with the modeled hemodynamic responses were computed to fit the observed BOLD-signal time course in each voxel for each participant using the general linear model with six regressors of interest. Six head-motion parameters, describing translation and rotation of the head, plus one constant term were included as nuisance regressors. In a second-level analysis, specific contrast images were brought forward into a random-effects analysis. The resulting statistical parametric maps were interpolated to facilitate comparison between conditions. Talairach coordinates were extracted using NeuroElf (neuroelf.net).

### Definition of statistical contrasts

We performed three sets of analyses on the images. The first one tested the main effect of partnering and the difference between the three partnered conditions (Leading, Following, and Mutual). The second one assessed the main effect and specificity of improvisation. The third set assessed the specific effect of improvising during partnered movements. Direct contrasts and conjunctions between conditions were performed at p < 0.05 corrected with the False Discovery Rate (FDR) and a cluster threshold of k = 20 voxels. In order to perform three-way comparisons, we computed contrasts between high-level contrasts and a conjunction of contrasts among the conditions (see below). These were run at p < 0.005 uncorrected, with a cluster threshold of k = 20 voxels (applied to the final statistical maps). We used NeuroElf to estimate the cluster-level correction of the uncorrected contrasts with Alphasim (family-wise error correction p<0.05), and found it to be lower than 20 voxels. All contrasts were balanced.

#### Effects of partnering

To identify brain regions associated with partnered movement, we examined the contrast [Leading + Following + Mutual] > [Solo + Alone]. We tested for the specificity of Leading, Following and Mutual among themselves after removing both basic motor effects (i.e., the non-partnered conditions, Solo and Alone) and partnering effects that all three conditions shared (i.e., the Partnership Conjunction), where the Partnership Conjunction is defined as [Leading > Non-Partnered] ∩ [Following > Non-Partnered] ∩ [Mutual > Non-Partnered]. Specifically, we used the following contrasts: 1) Leading: [Leading > Non-Partnered] > [Partnership Conjunction], 2) Following: [Following > Non-Partnered] > [Partnership Conjunction], and 3) Mutual: [Mutual > Non-Partnered] > [Partnership Conjunction].

#### Effects of improvisation

We tested the main effect of improvisation irrespective of partnership by examining the contrast [Leading + Solo] > [Mutual + Alone]. However, these pairs of conditions actually varied in two manners. One was with regard to improvisation, while the other was with respect to movement variability. The improvised conditions tended to have more variability in motion compared to the sequences used in the non-improvised tasks, which were fixed and repetitive. We therefore used the Following condition to disentangle this situation, since 1) movement variability in Following was similar to that during Leading and Solo, but 2) Following did not require improvisation on the part of the participant, as with Mutual and Alone. We conducted region-of-interest (ROI) analyses, in which ROI’s were defined as spheres of 5 mm radius centered on the peaks of the activations found in the improvisation contrast [Leading + Solo] > [Mutual + Alone]. Beta values were extracted from these ROI’s, and t-tests were conducted to determine if the betas for Following were significantly different from the mean betas for the improvised conditions, on the one hand, and the non-improvised conditions, on the other. We defined purely improvisation-related areas as those whose activity was significantly higher when participants *generated* novel motor sequences (i.e., Leading and Solo) compared with when they *executed* similar sequences without generating them (Following). In contrast, we defined areas related to movement variability as those whose activity was significantly higher when participants executed *variable* sequences (Following) compared with when they executed *repetitive* sequences (Mutual and Alone).

Next, we looked at brain areas that were involved in self-initiation of movement (Leading, Mutual, Solo, Alone) versus Following as the one externally-driven condition. We did this with the contrast [Leading + Solo + Mutual + Alone] > Following.

#### Effects of improvising with a partner

Finally, we wanted to explore if there was an effect of improvising with a partner compared to improvising solo, beyond the mere presence of skin-to-skin contact. Such an effect might reflect an interpersonal signalling strategy in leaders during joint action. First, we directly contrasted Leading and Solo. Next, to search for activity for Leading that was not attributable to either improvisational production (as in Solo) or physical contact with a partner (as in Following and Mutual), we performed the contrast [Leading > Mutual] > [Solo > Alone], and the conjunction [Leading > Solo] ∩ [Leading > Following].

## Results

### Behavioral data

Post-scanning questionnaires revealed a main effect of condition for all four questions, as follows: perceived difficulty (D): F(4,85) = 5.62, p < 0.001; performance quality (P): F(4,85) = 3.31, p = 0.014; impression of dancing: F(4,85) = 9.83, p < 0.001; and musical imagery: F(4,85) = 9.76, p = 0.001. The first two effects were due to an increased perception of difficulty and a decreased perception of performance quality for Leading (D 2.1, P 4.1) and Following (D 2.0, P 4.1) compared to the Alone condition (D 1.1, P 4.7), with Solo (D 1.6, P 4.5) and Mutual (D 1.5, P 4.5) sitting in between. In general, perceived difficulty was low and perceived performance quality was high for all tasks. Interestingly, conditions with more-variable motor sequences were perceived as more dance-like (Leading: 3.9, Following: 3.7, and Solo: 4.0) than conditions with repetitive motor sequences (Mutual: 2.5, Alone: 2.4). The improvised conditions elicited more musical imagery (Leading: 3.9 and Solo: 4.0) than the non-improvised conditions (Following: 2.4, Mutual: 2.1, Alone: 2.7).

### fMRI

#### Partnering

**Fig 2:**
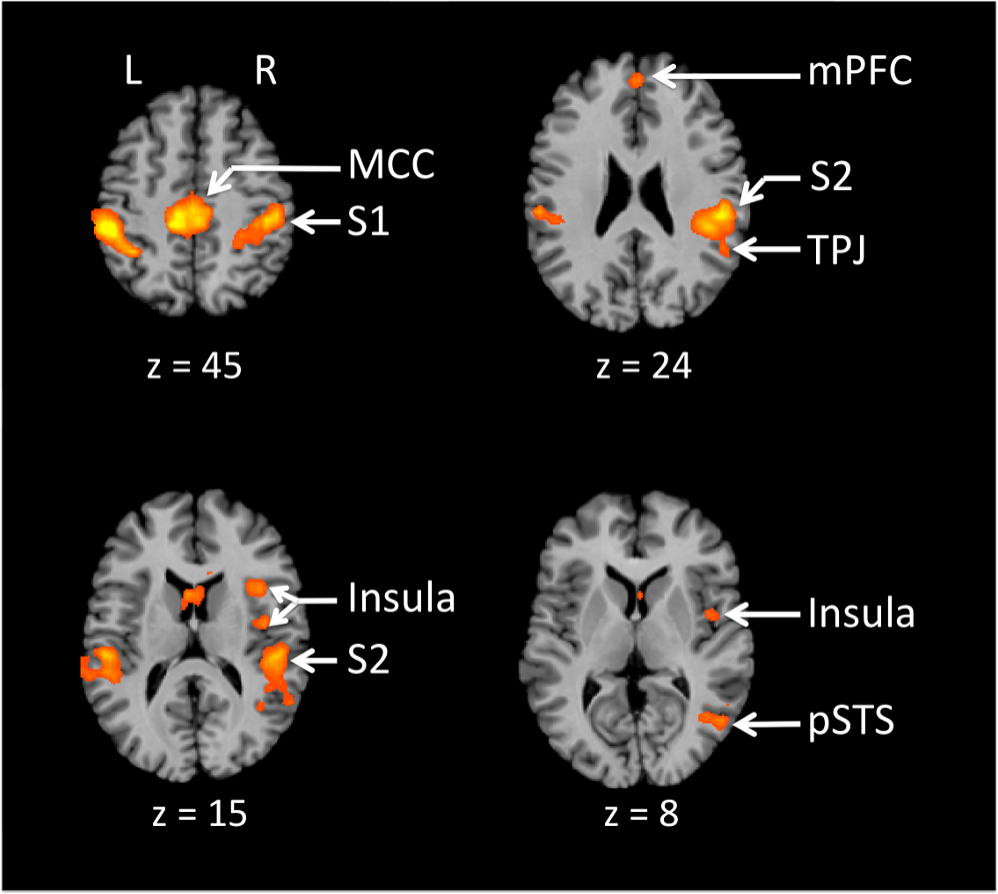
Main effect of partnering. Partnering was examined by contrasting the partnered conditions with the non-partnered conditions (Leading + Following + Mutual > Solo + Alone), with results reported at p < 0.05, FDR corrected, with a cluster threshold k = 20. The results in Figs 2–4 are registered onto a Talairach-normalized anatomical template MRI (the Colin brain). The Talairach z coordinate is shown below each slice. The left side of the slice is the left side of the brain. Abbreviations: MCC: middle cingulate cortex; mPFC: medial prefrontal cortex; pSTS: posterior superior temporal sulcus; S1: primary somatosensory cortex; S2: secondary somatosensory cortex; TPJ: temporo-parietal junction.

We examined the main effect of partnering by contrasting the three partnered conditions with the two non-partnered conditions (Fig 2, with Talairach coordinates in Table 1). As expected, we found strong activations in brain regions involved in tactile perception and proprioception, including the primary somatosensory cortex (S1), secondary somatosensory cortex (S2), and ventral thalamus. In addition, we found activity in limbic areas such as the midcingulate cortex (MCC) and anterior insula that are involved in orienting the body to cutaneous stimuli and in processing internal sensation. Finally, we observed activity in areas involved in the perception of dynamic social stimuli (pSTS) and mentalizing (mPFC and TPJ).

**Table 1:** Partnering. Talairach coordinates for the peak activations for the contrast “partnered versus non-partnered” (i.e., Leading + Following + Mutual > Solo + Alone), p < 0.05 with FDR correction. BA = Brodmann area, k = number of voxels, t = maximum t value, RH = right hemisphere, LH = left hemisphere. Abbreviations: aIPL, anterior inferior parietal lobule; FG, fusiform gyrus; IFG, inferior frontal gyrus; MCC, middle cingulate cortex; mPFC, medial prefrontal cortex; MT+/V5, motion area of the middle temporal region; S1, primary somatosensory cortex; S2, secondary somatosensory cortex; pSTS, posterior superior temporal sulcus; TPJ = temporo-parietal junction.

			TAL coordinates				
Areas	Hemisphere	BA	x	y	z	t (peak)	k
FRONTAL							
mPFC	RH	9	3	47	28	7.08	53
Insula	RH	13, 45	39	14	16	5.58	57
Insula	RH	13, 44	42	-7	16	5.47	55
mPFC	LH	10	-9	47	-5	5.43	22
IFG	RH	47	39	17	-11	5.15	29
PARIETAL							
S1	LH	2, 3, 7, 40	-48	-34	49	11.02	462
S1	RH	2, 3, 7, 40	45	-28	52	10.65	617
S2/aIPL	RH	40, 13	45	-28	19	10.07	293
MCC	LH	5, 31	-12	-31	46	9.37	338
S2/aIPL	LH	40, 13, 22	-51	-25	16	7.19	174
TEMPORAL							
MT+	RH	37, 39	51	-61	7	5.57	100
Fusiform	RH	37	48	-61	-8	5.50	36
TPJ ant	RH	40	51	-46	25	4.91	43
STS	RH	39, 22	39	-55	16	4.57	42
SUBCORTICAL							
Caudate	LH		-3	11	16	5.83	69
Thalamus	RH		12	-16	1	4.94	95
Claustrum	RH		33	-7	-5	4.40	31
CEREBELLUM							
Declive	RH		42	-67	-20	4.85	27
Culmen	RH		15	-52	-17	4.46	29

**Fig 3:**
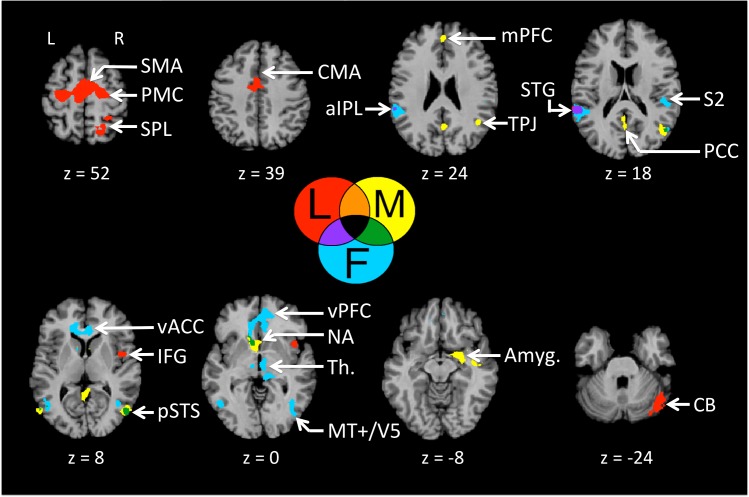
Specificity for Leading, Following, and Mutual. Neural specificity for Leading, Following and Mutual is shown, after removing both basic motoric effects (through subtraction of the non-partnered conditions Solo + Alone) and partnering effects, as seen in a conjunction of the three partnership contrasts: [Leading > non-partnered conditions] ∩ [Following > non-partnered conditions] ∩ [Mutual> non-partnered conditions]. The role-specific activations are color-coded as follows: Leading (red): [Leading > Non-partnered conditions] > [Partnership Conjunction]; Following (blue): [Following > Non-partnered conditions] > [Partnership Conjunction]; and Mutual (yellow): [Mutual> Non-partnered conditions] > [Partnership Conjunction]. The results are p < 0.005 uncorrected, with a cluster threshold k = 20. Abbreviations: aIPL: anterior inferior parietal lobule; MCC: middle cingulate cortex; MT+/V5, motion area of the middle temporal region; mPFC: medial prefrontal cortex; NA: nucleus accumbens; pSTS: posterior superior temporal sulcus; S1: primary somatosensory cortex; S2: secondary somatosensory cortex; TPJ: temporo-parietal junction.

Having looked at what the three partnered conditions shared, we next explored role-related effects by examining neural specificity for Leading, Following, and Mutual among themselves (Fig 3 and Table 2). Relative to the other two partnered conditions, Leading showed a clear profile indicative of motor planning and self-initiation of motor production. This included a series of brain areas involved in motor execution (primary motor cortex [M1]), motor planning (premotor cortex [PMC] and cingulate motor area [CMA]), bimanual coordination and internal initiation (supplementary motor area [SMA]), spatial navigation of the limbs (superior parietal lobule [SPL]), motor sequencing (inferior frontal gyrus [IFG]), error correction (lateral cerebellum), and the transformation of sensory information into temporally-organized motor actions (superior temporal gyrus [STG]), this latter of which overlapped with activations for Following.

**Table 2:** Specific activations for Leading, Following, and Mutual. Talairach coordinates for the peak activations for the contrasts “one partnered condition versus the conjunction of the three partnered conditions” (see Methods for details), p < 0.005 uncorrected. BA = Brodmann area, k = number of voxels, t = maximum t value, RH = right hemisphere, LH = left hemisphere. Abbreviations: ACC, anterior cingulate cortex; aIPL, anterior inferior parietal lobule; Amyg., amygdala; CMA, cingulate motor area; IFG, inferior frontal gyrus; M1, primary motor cortex; MFG, medial frontal gyrus; mPFC, medial prefrontal cortex; MT+/V5, motion area of the middle temporal region; PMC, premotor cortex; pSTS, posterior superior temporal sulcus; Put., putamen; S2, secondary somatosensory cortex; SMA, supplementary motor area; SPL, superior parietal lobule; STG, superior temporal gyrus; TPJ, temporo-parietal junction.

			TAL coordinates				
Areas	Hemisphere	BA	x	y	z	t (peak)	k
**Leading > Conjunction**							
FRONTAL							
M1/PMC	LH	4, 6	-21	-16	58	10.36	159
SMA	RH/LH	6	0	-13	52	8.42	370
M1/PMC	RH	4, 6	15	-16	55	7.42	147
IFG	RH	44, 13	45	2	7	5.93	37
CMA	RH/LH	24	0	-1	43	5.61	68
PARIETAL							
SPL	RH	40, 7	30	-43	55	5.35	76
TEMPORAL							
STG	LH	13,22	-54	-37	19	6.21	31
CEREBELLUM							
Tuber	RH		45	-64	-23	6.41	86
**Following > Conjunction**							
FRONTAL							
mPFC	RH/LH	10	-9	38	-5	5.45	45
ACC	RH/LH	24,32,33	-3	26	7	4.86	199
MFG	LH	6	-9	-22	58	4.11	52
MFG	RH	6	9	-19	61	3.54	20
PARIETAL							
aIPL/STG	LH	40	-54	-37	25	6.75	101
S2	RH	41	48	-28	19	4.67	20
aIPL/STG	RH	13	51	-43	22	4.33	20
TEMPORAL							
MT+/V5	LH	37	-45	-61	4	5.60	34
MT+/V5	RH	37,19	39	-58	1	4.96	27
STS	RH	39,19	51	-67	10	4.46	42
STS	LH	39	-48	-52	10	3.22	20
SUBCORTICAL							
Thalamus	RH/LH		3	-13	1	4.93	45
Thalamus	RH		9	-28	1	4.40	27
Accumbens	LH		-9	11	13	4.26	20
Accumbens	RH		3	2	10	3.59	22
**Mutual > Conjunction**							
FRONTAL							
mPFC	RH/LH	9	3	44	31	5.05	20
PARIETAL							
PCC	RH/LH	30, 23	0	-49	13	4.58	73
TPJ ant	RH	39	42	-52	25	4.31	27
TEMPORAL							
STS	RH	39, 19	48	-67	16	6.33	64
STS	LH	39, 19	-45	-61	16	3.87	29
SUBCORTICAL							
Put./Amyg.	RH		21	-1	-8	5.82	99
Accumbens	RH/LH		-6	14	4	4.89	69

In contrast to the heavily motoric profile for Leading, Following showed a far more sensory profile, indicative of a responsiveness to external signals coming from the leader, where these signals serve as cues to guide movement. This included areas involved in tactile perception and proprioception (S2 and the sensory thalamus), motion tracking and social motion perception (MT+/V5 and pSTS), sensorimotor mapping of self and other’s actions (anterior inferior parietal lobule [aIPL]), and the monitoring of external outcomes in relation to reward (ventral anterior cingulate cortex [vACC], extending into the ventral mPFC, as well as the caudate nucleus and nucleus accumbens). It is clear from these results that leading and following represent reciprocal specializations in the brain, with leading highlighting self-initiation of movement and following an external orientation toward sensory signals coming from the partner’s actions.

The profile for the Mutual condition was distinguished from both Leading and Following by the presence of enhanced activity in the mentalizing network, including the mPFC, posterior cingulate cortex (PCC), and TPJ. This network was also part of the partnering network shared between Mutual, Leading and Following (Fig 2), but was more strongly recruited during Mutual, as if mutual interaction required a higher degree of awareness of the thoughts and intentions of the partner. The Mutual condition also recruited limbic areas involved in emotion and reward, including the amygdala and the nucleus accumbens. Unexpectedly, there was no overlap between Mutual and Leading in the three-way comparison (i.e., no area that was more activated for both Mutual and Leading than Following). However Mutual activity overlapped with Following activity in several regions, such as the pSTS and the nucleus accumbens. Overall, this suggests that mutual interaction might be more of a form of mutual following than mutual leading.

#### Improvisation

**Fig 4:**
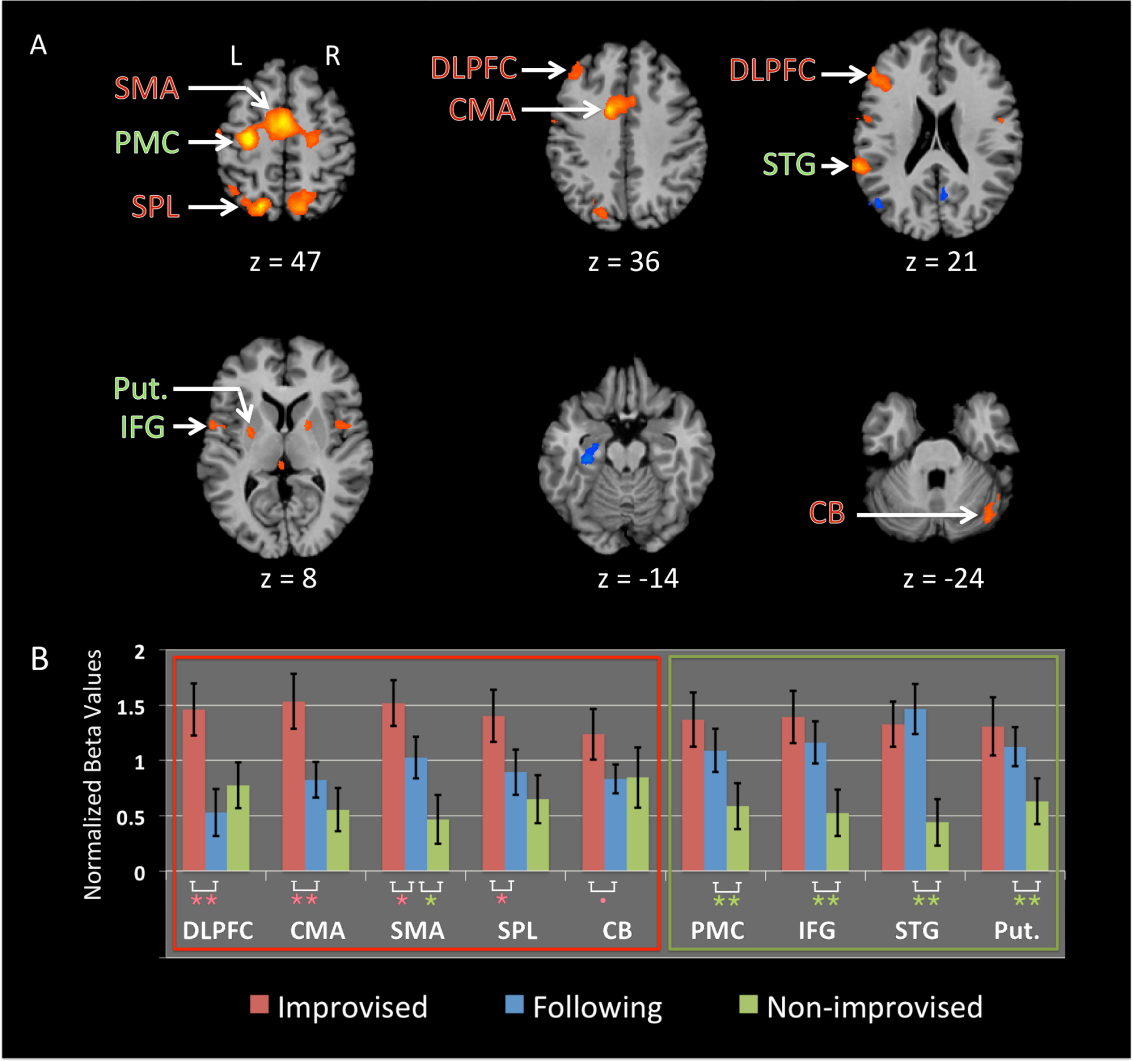
Effect of improvisation. (A) Whole-brain analysis of improvisation: Leading + Solo > Mutual + Alone. p < 0.05 FDR corrected with a cluster threshold k = 20. Orange = activation, and blue = deactivation. Based on the results of the ROI analysis presented in panel B, areas with red labels in this panel (and that are surrounded with a red box in panel B) are those that are more likely to be involved in improvisational generation of movement, whereas those areas with green labels (and that are surrounded with a green box in panel B) are more likely to be associated with movement variability, rather than improvisation. Abbreviations: CMA: cingulate motor area; DLPFC: dorsolateral prefrontal cortex; IFG: inferior frontal gyrus; PMC: premotor cortex; SMA: supplementary motor area; SPL: superior parietal lobule; STG: superior temporal gyrus. (B) ROI analysis of the improvisation areas in comparison with the Following condition. ** = p < 0.005, * = p < 0.05, • = trend (p = 0.057). Error bars are standard errors of the mean. Beta weights extracted from spheres of 5 mm radius are centered on the peak of the areas defined in the improvisation contrast in panel A (Leading + Solo > Mutual + Alone). Improvised (red): mean across Leading + Solo. Non-improvised (green): mean across Mutual + Alone. The means are averaged across both hemispheres for the bilateral areas (see Table 3).

We examined the main effect of improvisation independent of partnership by contrasting the improvised generative conditions (Leading and Solo) with their fixed-pattern counterparts (Mutual and Alone). For reasons explained in the Methods section, Following was excluded from this analysis. Improvisation activated a network of brain areas similar to that for Leading, including M1, PMC, SMA, CMA, IFG, STG and SPL (Fig 4A and Table 3). Additional improvisation areas not found in Leading included the left dorsolateral prefrontal cortex (DLPFC) and bilateral putamen.

**Table 3:** Improvisation and self-initiation. Talairach coordinates for the peak activations and deactivations for the contrast “improvised versus non-improvised” (i.e., Leading + Solo > Mutual + Alone), p < 0.05 with FDR correction. Also included are the coordinates for the peak activations for the contrast “self-initiated versus externally-triggered” ([Leading + Solo + Mutual + Alone] > Following), p < 0.05 with FDR correction. BA = Brodmann area, k = number of voxels, t = maximum t value, RH = right hemisphere, LH = left hemisphere. Abbreviations: CMA, cingulate motor area; DLPFC, dorsolateral prefrontal cortex; IFG, inferior frontal gyrus; M1, primary motor cortex; PHC, parahippocampal gyrus: PMC, premotor cortex; SMA, supplementary motor area; SPL, superior parietal lobule; STG, superior temportal gyrus; TPJ = temporo-parietal junction.

			TAL coordinates				
Areas	Hemisphere	BA	x	y	z	t (peak)	k
**Improvisation**							
FRONTAL							
M1/PMC	LH	4, 6	-27	-16	52	13.13	168
SMA	RH/LH	6	0	-4	52	8.58	513
CMA	LH	24, 32	-12	5	37	8.19	211
M1/PMC	RH	4, 6	18	-22	55	6.71	114
DLPFC	LH	8, 9, 46	-48	32	31	6.01	105
PMC	LH	6, 22	-48	-1	28	5.71	41
IFG	RH	44, 22, 6	45	2	7	5.59	49
IFG	LH	22, 6	-54	5	4	5.25	49
PARIETAL							
SPL	LH	7	-18	-70	49	9.05	121
SPL	RH	7	15	-64	55	7.63	127
SPL	LH	7, 40	-39	-55	52	4.38	23
TEMPORAL							
STG	LH	22	-60	-40	19	7.41	67
SUBCORTICAL							
Thalamus	RH/LH		0	-16	16	5.64	33
Putamen	RH		18	2	10	4.90	20
Putamen	LH		-24	-7	10	4.63	22
CEREBELLUM							
Declive	RH		39	-70	-20	5.20	40
**Self-initiation**							
FRONTAL							
DLPFC	LH	9	-42	32	37	8.44	60
PARIETAL							
SPL	RH	7, 19	6	-82	49	5.87	77
SPL	LH	7, 19	-12	-82	46	6.65	198

The improvised conditions differed from the non-improvised conditions not just in creative generation but in movement variability as well (see the Methods section). We therefore used the Following condition as a means of disentangling these two effects, since its movement variability was similar to that of the improvised conditions, but it did not require the participant to improvise at the generative level. Brain regions that were engaged more during improvised movements than during Following are likely associated with movement generation. Brain regions that were engaged more during Following than the non-improvised conditions are likely associated with movement variability.

Those areas that were most strongly associated with improvisational movement generation (Fig 4B, red box) were the SMA, SPL, CMA DLPFC, and lateral cerebellum. These were areas in which the improvised conditions showed significantly greater activity than Following (marginally significant for the cerebellum). In contrast, areas more strongly associated with movement variability than creative generation per se (Fig 4B, green box) were the PMC, IFG, putamen, and STG, where Following showed significantly greater activity than the non-improvised conditions, but did not differ from the improvised conditions. Finally, the whole-brain analysis contrasting self-initiated movements that are either improvised (Leading and Solo) or performed by memory (Mutual and Alone) to externally-initiated movement (Following) gave rise to activity in the SPL and the left DLPFC (the coordinates are presented in Table 3 under “self-initiated activation”).

#### Improvising with a partner

We sought brain areas associated with signalling movement intentions to a partner by comparing the Leading and Solo conditions, since both are improvised and only differ in partnership. The Leading > Solo contrast showed the same set of brain areas that came up in the partnership contrast, with no additional areas showing up (see Table 4; the insula and mPFC were present only at a more liberal threshold). To further explore whether there was activity that was specific to leading within these partnership areas or if the areas were fully shared with the other partnered conditions, we performed the contrast [Leading > Mutual] > [Solo > Alone] as well as the conjunction [Leading > Solo] ∩ [Leading > Following]. No areas were present in either analysis, even at a more liberal threshold. Overall, this indicates that, at least within the context of the present experiment, Leading is simply the additive combination of solo improvisation and partnering, with no indication of an interaction effect.

**Table 4:** Joint improvisation. Talairach coordinates for the peak activations and deactivations for the contrast “Leading > Solo”, p < 0.05 with FDR correction. BA = Brodmann area, k = number of voxels, t = maximum t value, RH = right hemisphere, LH = left hemisphere. Abbreviations: MCC, middle cingulate cortex; S1, primary somatosensory cortex; S2, secondary somatosensory cortex.

			TAL coordinates				
Areas	Hemisphere	BA	x	y	z	t (peak)	k
S1	RH	2,3,4,5,40	42	-34	58	9.17	283
S1	LH	3,40	-48	-34	46	8.73	210
MCC	LH	31	0	-22	46	7.75	79
S2	RH	13,40	45	-34	22	6.08	69

## Discussion

The capacity for joint action is a critical part of the social phenotype that permits humans to engage in cooperative actions. The majority of such joint interactions involve a balance between the more dominant pattern of leading and the more receptive pattern of following. In addition, one class of such interactions involves direct physical contact between the participants, spanning from sexual intercourse to group dancing. We have reported the results of the first experiment to examine the neural basis of leading and following during a situation of joint improvisation with direct haptic contact, employing a novel two-person scanning arrangement. Consistent with our predictions, leading was characterized by a motoric profile that reflected the role of leaders in motor planning, navigation, and the conveyance of forces to a partner. Following, by contrast, was associated with a far more sensory profile that reflected the role of followers in responding to the directional cues of a leader and in tracking the leader’s movements. The mutual condition–in which we attempted to eliminate the role asymmetry present in the leading and following conditions by employing pre-learned movement patterns within the context of a symmetric interaction–had an activation profile similar to following, suggesting that both partners may have mutually followed one another. Finally, the analysis of improvisation revealed the importance of both premotor and working-memory areas for improvised movements compared to fixed movement patterns. It also permitted a disambiguation of which improvisation areas were associated with movement generation, compared to movement variability per se. Overall, these results present a first look at the brain systems important for leading and following in a situation of joint action with direct haptic contact.

### Partnering

Joint action is characterized by a continuous interaction between partners. As expected given the haptic interaction occurring between partners in our paradigm, the analysis of partnering revealed activity in cortical areas that respond to somatosensory and proprioceptive stimulation, including the primary and secondary somatosensory cortices. Other areas included the pSTS, MCC, anterior and posterior insula, TPJ, and mPFC. The pSTS is a multimodal area [40] that processes dynamic social stimuli, including biological motion [41], implied human motion [42], facial expression [43], vocal prosody [44], eye movement, social gaze [45,46], and even animacy detection [47]. It is also involved in haptic identification of facial expression [48]. We found the pSTS to be more active during partnered than non-partnered movement, but most especially during the mutual and following conditions, where participants relied more heavily on haptic cues coming from their partner’s movements. Haptic coupling with a partner can be seen as a form of dynamic social communication in which haptic cues are used to convey a partner’s intentions.

The posterior MCC is commonly activated in studies that use tactile stimuli (e.g., [49,50]). It mediates response selection and body orientation toward somatic stimuli, and is functionally connected with the posterior insula [51,52]. The posterior MCC could therefore have played a role in the orientation of the hands in response to tactile cues coming from one’s partner. For this reason, we predict that it would be involved in skeletomotor orientation during whole-body dance partnering. Related to the MCC, the posterior insula is stimulated by muscle activation during exercise [53], and is associated with interoception, the perception of emotional salience, and self-body consciousness. Hence, the involvement of the posterior insula in partnering might relate to increase reliance on internally-salient sensation and self-body awareness. The anterior insula, another area activated in the partnering contrast, is associated by subjective feeling and also trust with a partner [53,54]. It is thought to mediate interaction between brain regions that are externally salient and those that are internally salient [55,56], and so it could play a key role during social interactions.

Finally, all of the partnered tasks elicited activity in the TPJ and mPFC. These areas are core components of the mentalizing network, which is responsible for the ability to understand the mental states of others, to predict their intentions, and to think about social attributes of the self and others [57–60]. This network is activated both when observing and engaging in social interactions [16,61]. Its presence in our partnership contrast might reflect the mentalizing about a partner’s intentions that occurs during a socially-interactive task, as compared to an action done on one’s own.

### Leading and Following

Having established the basic network involved in haptic interaction between partners in a situation of joint action, we wanted to explore brain areas that were specific for either leading or following during partnered movement. As predicted, leading showed a motoric profile related to movement planning, self-initiation, and spatial navigation, whereas following showed a sensory profile related to haptic awareness, motion tracking, and social perception. Leading’s emphasis on motor activations is consistent with behavioral studies showing that leading, compared to following, involves a greater degree of movement control and a reduced degree of movement variability, both of which aim to achieve stable partnering of movement [4,62].

The brain network for leading was highly similar to that revealed for leading in studies of auditory finger tapping [3] and musical duetting [9,10], as well as that of the initiator in studies of reciprocal imitation of people’s actions [12,14]. This network is associated with self-initiated action, decision making, self-prioritization, multi-limb coordination, and motor control. It includes the PMC, SMA, CMA, and cerebellum. In addition, our improvisational task required that the leader generate novel movement sequences throughout the task. Activation of the right IFG (BA 44) has been associated with the production of novel movement sequences, while controlling for rules maintenance, as seen in studies of improvisation during piano performance [27,28]. Moreover, activity in the IFG has been shown to be correlated with the perceived influence of the self on a virtual partner in a joint task [3], which is consistent with a role in leading. Finally, leading showed activity in the SPL, an area involved in spatially-oriented motor planning and attention that integrates visual, proprioceptive, somatosensory, and auditory information [63–65]. Lesions to the SPL have been shown to lead to impairments in tactile search [66]. A medial part of the SPL, located in the precuneus, has been shown to be activated by spatial navigation of the lower limbs in a non-partnered dance-like experiment [67], and so likely plays a similar role for the upper-limb movement in the present experiment. The SPL is likewise engaged in studies of gestural imitation and pantomime using the upper limbs [68,69]. Leading requires an exploration and representation of movement patterns in space in order to create a coordinated “dance” between the partners. However, a similar area within the SPL was shown to be engaged during a leading task without a spatial component [3], which was attributed to the integration of self and other information.

There is little literature regarding the neural basis of following. In fact, previous studies examining leading and following reported no activation for following compared to leading [3] or activations that were due to deactivations during leading [12]. The brain network that was engaged during our following task had a strong sensory orientation. This included areas involved in the processing of somatosensory and proprioceptive stimuli (the sensory thalamus and S2, [70–73]), motion perception (MT+/V5, [74,75]), and the perception of dynamic social stimuli (pSTS, [76]). Such areas support a follower’s enhanced receptivity to haptic motion-cues coming from the leader. This is consistent with the enhanced receptivity to visual cues that occurs during visual imitation of a partner’s movement [12,77]. Other areas associated with following included the ventral ACC and mPFC. These areas have been implicated in reward and punishment, as well as in assigning value to the outcome of an action [57]. More specifically, the ventral mPFC plays a role in monitoring the performance of one’s own actions that are initiated by someone else [78]. This should thus be an important system for monitoring performance during following, in other words when one is not deciding on the action to be performed. It is notable that patients with damage to the ventral mPFC are less likely to emerge as followers when asked to respond to a leader’s offer [25].

The contrastive networks engaged by leading vs. following highlight the complementary nature of these roles during joint action. While leading requires an internal orientation to movement execution, as related to self-initiation of movement (PMC and SMA), following requires an external orientation to movement execution, as related to responsiveness to the movement cues coming from a partner. While leading requires the planning and generation of movement sequences (IFG) occurring in a spatially-patterned manner (SPL), following requires a heightened awareness of sensory cues coming from leader (sensory thalamus and S2) and a system to track motion-cues from the leader (MT+/V5 and pSTS). Finally, the CMA (dACC), which was activated during leading, plays a role in monitoring performance during self-initiated actions, whereas the ventral ACC and mPFC, which were activated during following, play a role in monitoring performance during externally-triggered actions.

As mentioned in the Introduction, we acknowledge that during true social interactions, both leading and following are tasks that require individuals to adapt to one another in a bilateral fashion, and that neither activity involves unilateral conveyance (leading) or receipt (following) of forces [6–8]. However, due to the simplicity of our task, it is unlikely that the follower needed to improvise a motor plan, or that the leader needed to adapt his motor plan to the follower’s behavior. This was not observed by the experimenter while interacting with the participants.

### Mutual partnering

Although our overarching goal in the present study was to identify the brain areas that differentiated leading from following, we also wanted to explore a related facet of joint action in which the partners’ roles were symmetric. An important question that we wanted to address was whether an egalitarian action–in which partners share symmetrical roles–is more similar to a process of mutual leading or one of mutual following. While the brain activity for the mutual condition was, in fact, different from both leading and following, it showed a profile much closer to following than leading, suggesting that mutual interaction might be a form of reciprocal following. This was seen particularly with reference to the overlapping activity between mutual and following in the pSTS. It should be pointed out, however, that this profile of mutual following might be dependant on the pre-learned nature of our task. We would speculate that a condition in which the partners’ movements were jointly improvised might in fact be closer to a situation of mutual leading than mutual following. However, even in such a situation, there might be bouts in which the jointly improvising partners would achieve a state of “togetherness” (as described in [36]), so that both leading and following would come equally into play, as related to both self-focused motor planning and other-focused sensory receptiveness. We would additionally hypothesize that the core network for mutual interaction outlined here (i.e., areas for mentalizing and social processing) would be present, regardless of the type of mutual interaction.

While the mentalizing network was associated with partnering in general, it was preferentially engaged during mutual interaction. This suggests that our interpretation of this condition as reciprocal following might be underlain by ongoing mentalizing about the intentions of the partner as a strategy for achieving this reciprocity. The TPJ, mPFC and PCC were more activated during the mutual condition than both leading and following. The mPFC, in particular, has been shown to be involved in reasoning about others and the self in social contexts [79]. It is activated, for example, when our own actions have consequence for others in joint-action settings [80]. The fact that both self- and other-strategies have to be taken into account during the mutual condition may explain the greater involvement of the mPFC in mutual compared to leading and following. The TPJ was shown previously to be activated in a joint-action paradigm in which both individuals shared the same role [16], where the authors attributed TPJ activity to the perception of agency in an ambiguous situation. In our mutual task, participants were instructed that the speed and amplitude of the motion should arise by mutual agreement, whereas during the leading and following conditions these parameters were determined by the leader only. For this reason, agency would be more ambiguous during the mutual task, which might explain the additional engagement of mentalizing areas such as the TPJ, PCC and mPFC during this condition compared to the other two partnered conditions. The PCC plays a role in self-related processing [76], and is believed to balance internal and external foci of attention [81], which could be a key facet of the mutual task, since individuals have to focus on themselves but at the same time pay attention to their partner. In addition, there is a lesser need to predict and integrate another’s actions into our own motor plan during symmetric, as compared to complementary, interactions [61,82], as suggested by the reduced activation in the anterior IPL during mutual interaction, compared to leading and following. This area supports action coordination during interpersonal interactions, playing a role in the prediction of self and other sensory experiences and the integration of such predicted experiences with motor programs [83]. Overall, the differential involvement of mentalizing areas in the mutual condition, compared to leading and following, is consistent with previous research on the activity of this network in joint-action settings, such as when one’s own behavior affects joint performance or when partners’ roles are shared versus complementary.

The final class of brain areas specific for mutual interaction comprised areas involved in emotion and reward, including the amygdala and nucleus accumbens. The amygdala is an area important for emotional and motivational functions that is also thought to play a role in social interaction. It is involved in making the decision to cooperate with others [84,85], as would occur in our mutual task, with its bidirectional interactions. The activation in a reward center like the nucleus accumbens in our mutual task is perhaps the most interesting aspect of the results, which might suggest that “keeping together in time” with other people [86] is associated with a rewarding feeling of pleasure [87]. The nucleus accumbens is one of the key reward centers of the brain [88], playing a general role in cognition and action [89]. Its activity has been shown to be greater for social rewards than for non-social rewards, such as drugs [90,91]. Overall, the most cooperative, reciprocal and egalitarian of our joint-action conditions was associated with a neural signature of pleasure, suggesting that this form of mutuality is perceived as socially rewarding by its participants. This is in line with numerous studies showing that interpersonal interactions and mutual contingency between individuals engage reward centers, and do so more during cooperative than competitive interactions [92–94]. It is thus not surprising that activation in the social, mentalizing and reward networks would increase in parallel in our three partnered tasks, being lowest in the most self-focused condition (leading) and the highest in the most reciprocal one (mutual), with following being intermediate.

### Improvisation

Our study involved patterned movement of the limbs that occurred in an improvised manner, and thus might serve as a neuroimaging model of dance improvisation. Improvisational movement of the hands, when contrasted with performing fixed and pre-learned movement patterns, led to activation in two general types of areas, namely premotor areas that may be specific to the domain of bimanual movement and working-memory areas that may generalize across movement domains. The network of areas seen for improvisation in our study was highly similar to that found in previous studies of musical improvisation [26–28,30–32] and random sequence generation [28,30,35], which includes the dorsal PMC, SMA, CMA, IFG, DLPFC, SPL, STG and putamen.

A more detailed analysis of the results allowed us to disentangle the function of these areas in improvisation compared with the production of sequence complexity/variability per se. We defined improvisation-related areas as those whose activity was significantly higher when participants *generated* novel motor sequences (i.e., leading and solo) compared with when they *executed* similar sequences without generating them (following). This network comprised three groups of areas. First, the CMA and cerebellum seemed not to be related to movement complexity, as there was no difference in their activity between following and the non-improvised conditions. Instead, the CMA is involved in decision making, willed action, voluntary selection, and sequence generation [12,27]. Second, the SMA seems to play a role in both processes, as its activation during following was significantly higher than during the non-improvised conditions, but still less than during leading and solo. The SMA has been involved in many different processes, including self-triggered actions that are guided by an internal model or external cues [95], and the simulation/prediction of actions or events [96]. Thus, it could be more important for coordinating complex motor sequences than simple ones, and even more so if these sequences are internally generated online.

The third group of improvisation-related areas includes the DLPFC and SPL, where following was significantly lower than the non-improvised conditions. These areas were more activated during self-initiated movement than externally-triggered movement, regardless of whether the movement was improvised or performed from memory, but were still more active during improvisation. The DLPFC is activated when at least one parameter of the action is self-initiated [97–99]. It plays a role in the monitoring of information in working memory [100], attention during selection of action [12,28], suppression of unwanted responses, and the maintenance of the global motor plans [30]. It is more activated during complex than simple improvisation [30], as seen in our results. Regarding the SPL, whose role was discussed above, we suggest that, in our study, it was responsible for the voluntary exploration and control of the limbs in space during self-initiated actions. The more that there was a need for spatial exploration (improvisation), the more the SPL was activated. Most of brain areas associated with improvisation comprised premotor areas, consistent with a general finding in the literature that improvisation is mediated in large part by augmented activity in domain-specific motor-planning areas. Likewise, the association of the DLPFC with improvisation (rather than sequence complexity) is consistent with much published work on improvisation [28,30], where this area is thought to allocate domain-general resources for working memory.

Another set of regions that was more activated during improvised compared to non-improvised movement showed no difference in activity from following, therefore suggesting that they were associated with sequence complexity, rather than the generative component of improvisation. This included the IFG, dorsal PMC, putamen, and STG. While all of these areas have been shown to be involved in improvisation in previous studies [27,28,31], those studies only contrasted improvisation with the performance of pre-learned sequences, whereas we were able to control for both improvisation and movement variability in our analysis. The IFG is involved in sequencing, particularly with respect to the integration of rules or goals stored in working memory [16,27]. Our results support the involvement of the IFG in the execution of novel sequences, but not necessarily in the internal generation of those sequences.

A similar account can be given for the dorsal PMC and STG, which were more activated during the production (but not necessarily the generation) of richer and more-variable sequences. The PMC plays a role in the selection of movements, either spatially or temporally [30,32]. It is involved in the complexity of movement that emerges from coordination between multiple effectors [101], such as bimanual coordination [102,103]. In addition, it shows greater activity with increasing motor difficulty [102,104]. The dorsal PMC receives information from a dorsal portion of the STG [105] (also called area Spt, [106]). This multisensory area [107] deals with the transformation of sensory information into temporally organized motor actions [105], and has previously been implicated in musical improvisation [30,31]. Finally, the putamen is involved in sequence production, not least in sequence learning [108], such as that which underlies the imitative learning of song sequences in songbirds [109]. Not only is the putamen involved in internally-guided movement [110,111] and the generation of internal representations on external stimuli [95,112], but it is known to be modulated by movement complexity [113,114] and action selection [115,116]. Overall, the association of movement complexity in our improvised tasks with the IFG, PMC, STG and putamen fits well with the known function of these areas in rich movement sequencing.

We acknowledge that the improvisations in our study were very simple and that they might be closer to random generation than genuine improvisation. Even though studies of pseudorandom generation of responses have highlighted a network similar to the one used during true improvisation [28,30,35], further research is needed to explore more dance-like improvisations than the ones that were examined in the present study.

### Improvising during joint action

We sought brain areas associated with signalling movement intentions to a partner by comparing the Leading to Solo. The results suggest that leading may be reducible to solo improvisation done with a partner. The direct contrast between the leading and solo conditions revealed nothing more than those exact areas that appeared in the partnership contrast (i.e., S1, S2, and MCC, as well as insula and mPFC at a slightly more liberal threshold), suggesting that leading in our experimental paradigm was nothing more than the additive combination of improvisation and partnering. This conclusion was supported by the fact that the contrast [Leading > Mutual] > [Solo > Alone] and the conjunction between [Leading > Solo] with [Leading > Following] failed to demonstrate activity, even at a more liberal threshold. Therefore, we were not able to identify any brain area that would be indicative of the leader signalling intentions to his/her partner, as has been shown in behavioral studies of leading during joint-action tasks [4,5]. A possible explanation for this paradoxical finding is that our relatively simple task did not place sufficiently strong demands on the leader. Additional studies using more-complex interactions will be required to address this issue.

### Limitations

We were limited in our ability to measure behavioral performance during task production in the scanner due to an absence of MRI-compatible technologies like motion capture and electromyography. In spite of training the participants very thoroughly to match movement variation across conditions and despite the experimenter verifying in real time that this was indeed the case, we have no quantitative indicator of task performance. However, the motoric brain profile that we observed for leading–compared to following and mutual–would suggest that a difference in muscle force or motion between leading and following or mutual might not represent an artifactual difference between conditions but instead an indicator of the mechanistic nature of leading. We also note that our results of the leading task are concordant with previous paradigms that used more-restricted motor performance and interaction ([3] had no results for following). Further research combining fMRI with MRI-compatible versions of EMG or motion capture will be needed to further explore these effects.

## Conclusions

Using a novel two-person fMRI scanning arrangement, we elucidated for the first time neural differences between the motor-driven task of being a leader and the sensory-driven task of being a follower during a situation of joint improvisation with direct haptic contact. The results shed light not only on the complementary features of leading and following, but on the neural basis of improvisation as well. We found that performing partnered hand movements activated somatosensory as well as social networks. Leading such movements principally activated a motor network involved in motor planning, spatial navigation, and monitoring self-initiated action. In a complementary fashion, haptically following partnered movements engaged areas that monitor externally-triggered action as well as sensory-oriented areas that process somatosensation, motion perception, and the perception of dynamic social stimuli. In contrast to the asymmetry of leading and following, engagement in a more symmetric and mutual interaction increased activity in mentalizing areas and regions involved in social reward. We observed that dance-like improvisation engaged a similar network to musical improvisation or random sequence generation. Moreover, we were able to dissociate a network devoted to improvisation–such as would be engaged in internal sequence generation, decision making, and willed action–from a network involved in sequence variability and movement complexity.

Haptic contact has been a neglected topic in the neuroscience of social interaction. Our study unites haptic contact with the topic of joint action, and by doing so highlights the importance not only of social touch but of the reciprocal exchange of forces necessary for joint cooperative actions of all types.

## Supporting information

S1 Video Example of the movement task for the Following, Leading, Solo, Mutual, and Alone conditions. The movements seen during Following, Leading, and Solo are improvised, whereas the three patterns seen in the Mutual and Alone conditions are pre-learned. The video shows the tasks performed in the supine position, as in the MRI scanner. However, in the MRI scanner, participants’ forearms were strapped to the side of their body.
(MP4)

S1 Table Framewise displacement. Mean (standard deviation) of the relative framewise displacement (Siegel et al., 2014) for each participant and each scan, giving an estimate of the amount of head motion (in mm).
(DOCX)
