# Supplementary material for: Taking two to tango: fMRI analysis of improvised joint action with physical contact
Source: PLoS One. 2018 Jan 11;13(1):e0191098. doi: 10.1371/journal.pone.0191098 (PMC5764359; doi:10.1371/journal.pone.0191098)
Supplement: S1 Table — Mean (standard deviation) of the relative framewise displacement (Siegel et al., 2014) for each participant and each scan, giving an estimate of the amount of head motion (in mm). (DOCX) [file pone.0191098.s002.docx]

**S1 Table. Framewise displacement.** Mean (standard deviation) of the relative framewise displacement (Siegel et al., 2014) for each participant and each scan, giving an estimate of the amount of head motion (in mm).

|  | S01 | S02 | S03 | S04 | S05 | S06 |
| --- | --- | --- | --- | --- | --- | --- |
| scan 1 | 0.11 (0.08) | 0.07 (0.05) | 0.12 (0.09) | 0.09 (0.12) | 0.09 (0.08) | 0.10 (0.09) |
| scan 2 | 0.18 (0.10) | 0.11 (0.07) | 0.14 (0.10) | 0.13 (0.11) | 0.10 (0.09) | 0.13 (0.10) |
| scan 3 | 0.20 (0.12) | 0.16 (0.08) | 0.14 (0.08) | 0.16 (0.08) | 0.11 (0.09) | 0.12 (0.10) |
|  |  |  |  |  |  |  |
|  | S07 | S08 | S09 | S10 | S11 | S12 |
| scan 1 | 0.07 (0.05) | 0.15 (0.21) | 0.24 (0.27) | 0.05 (0.05) | 0.08 (0.06) | 0.16 (0.14) |
| scan 2 | 0.08 (0.05) | 0.15 (0.16) | 0.22 (0.23) | 0.06 (0.04) | 0.11 (0.08) | 0.09 (0.05) |
| scan 3 | 0.07 (0.05) | 0.12 (0.11) | 0.28 (0.31) | 0.09 (0.06) | 0.12 (0.07) | 0.10 (0.06) |
|  |  |  |  |  |  |  |
|  | S13 | S14 | S15 | S16 | S17 | S18 |
| scan 1 | 0.14 (0.12) | 0.10 (0.18) | 0.10 (0.07) | 0.20 (0.19) | 0.14 (0.09) | 0.19 (0.22) |
| scan 2 | 0.17 (0.12) | 0.07 (0.08) | 0.11 (0.07) | 0.22 (0.18) | 0.14 (0.10) | 0.19 (0.25) |
| scan 3 | 0.16 (0.13) | 0.07 (0.06) | 0.13 (0.11) | 0.22 (0.17) | 0.13 (0.09) | 0.32 (0.46) |
